# Supplementary material for: FUS Alters circRNA Metabolism in Human Motor Neurons Carrying the ALS-Linked P525L Mutation
Source: Int J Mol Sci. 2023 Feb 6;24(4):3181. doi: 10.3390/ijms24043181 (PMC9968238; doi:10.3390/ijms24043181)
Supplement: Supplementary file 1 [file ijms-24-03181-s001.zip › ijms-2105966-SI.pdf]

Figure S1

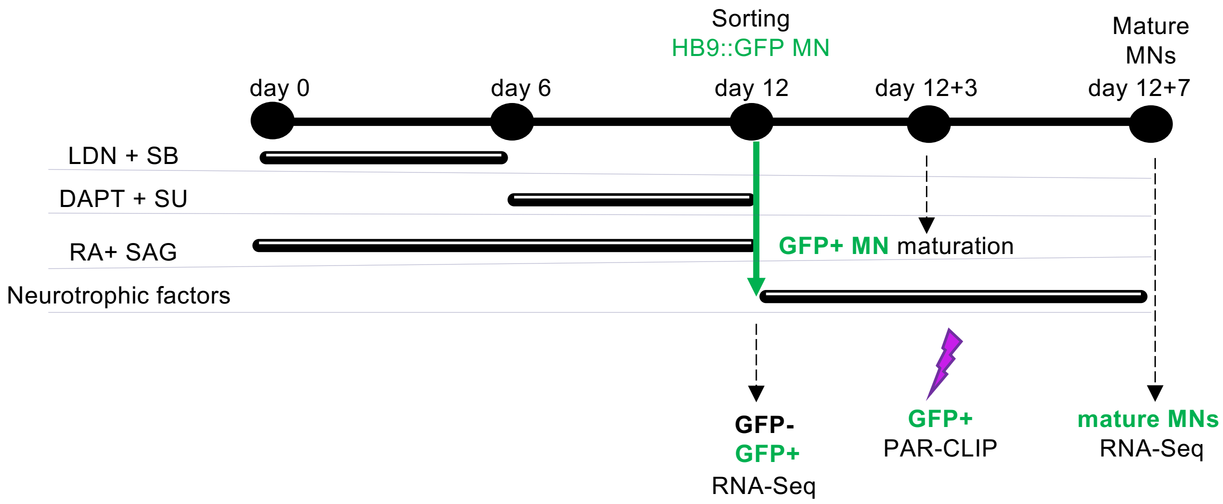

Figure S1. (Related to Figure 1) In vitro differentiation of iPSCs toward MN fate. Schematic representation of the differentiation protocol as described in De Santis et al., 2017. The medium and factors used along the timeline (day) of differentiation are indicated. Black and white thick lines depict the duration of the different treatments. iPSCs carry GFP reporter gene under the MN specific HB9 promoter and the expression of this reporter is indicated by green mark. At day 12 cells were FACS-sorted thanks to the expression of GFP reporter and GFP- and GFP+ populations were subjected to RNA-Seq (De Santis et al., 2017). Part of GFP+ cells were replated for 7 additional days of maturation (12+7) and then collected for RNA-Seq (mature MNs; De Santis et al 2017). The PAR-CLIP public data (De Santis et al., 2019) were produced using cells at 12+3 stage as reported in the scheme.

Figure S2

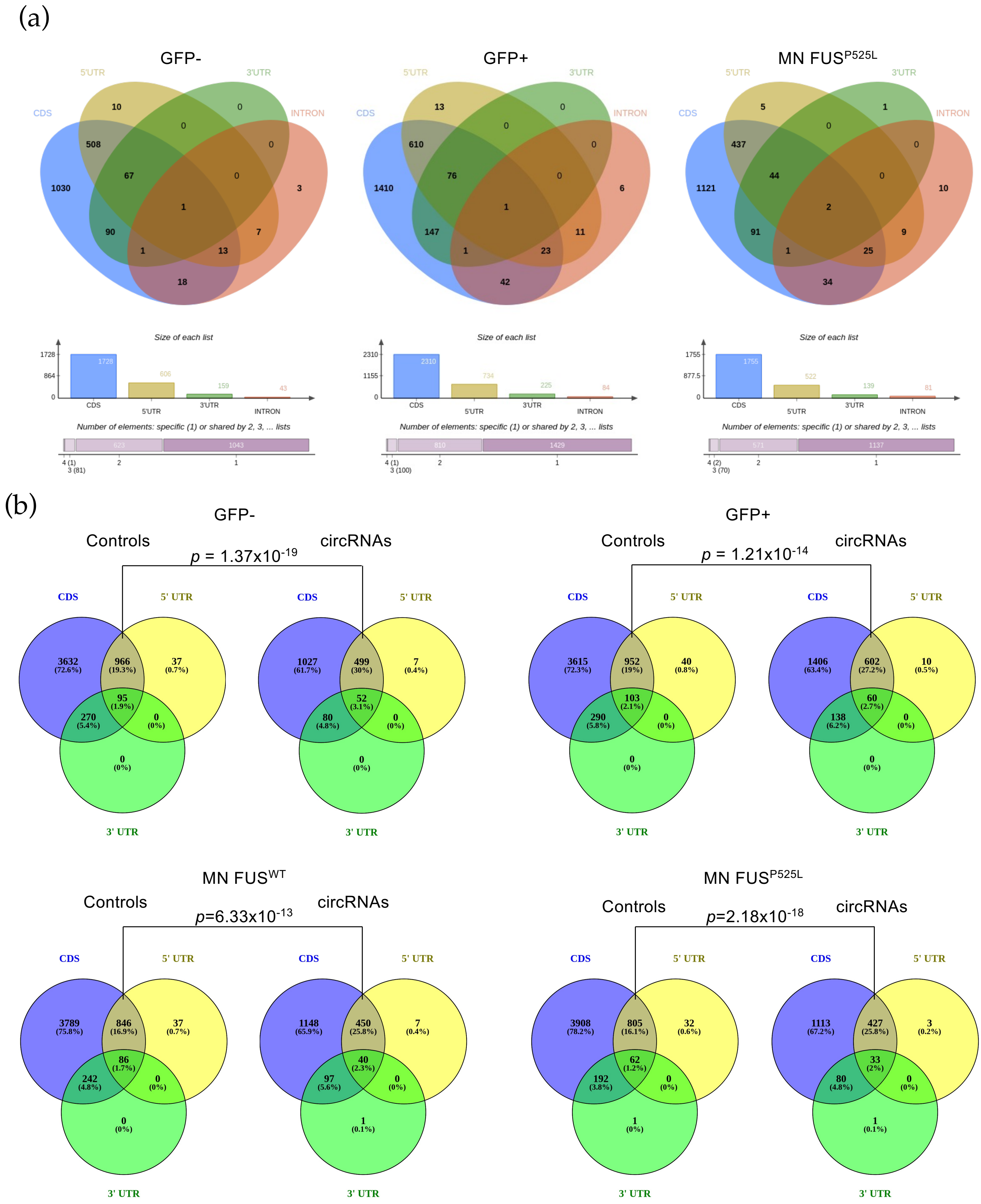

Figure S2 (related to Figure 1). CircRNA localization in host genes (a) Venn diagrams and bar charts showing the protein-coding gene regions occupied by the circRNAs detected in GFP-, GFP+ and MN FUS^{P525L} samples. (**b**) Venn diagram showing, for each cell type, the localization of non-intronic circRNAs and faux random circRNAs (controls) with respect to 5' UTR, 3'UTR and coding region of protein-coding transcripts. P-value was calculated by performing chi-squared test comparing the number of real and faux circRNAs spanning both 5'UTR and CDS regions. CDS: coding sequence; 5'UTR: 5' untranslated region; 3'UTR: 3' untranslated region; INTRON: intronic region.

Figure S3

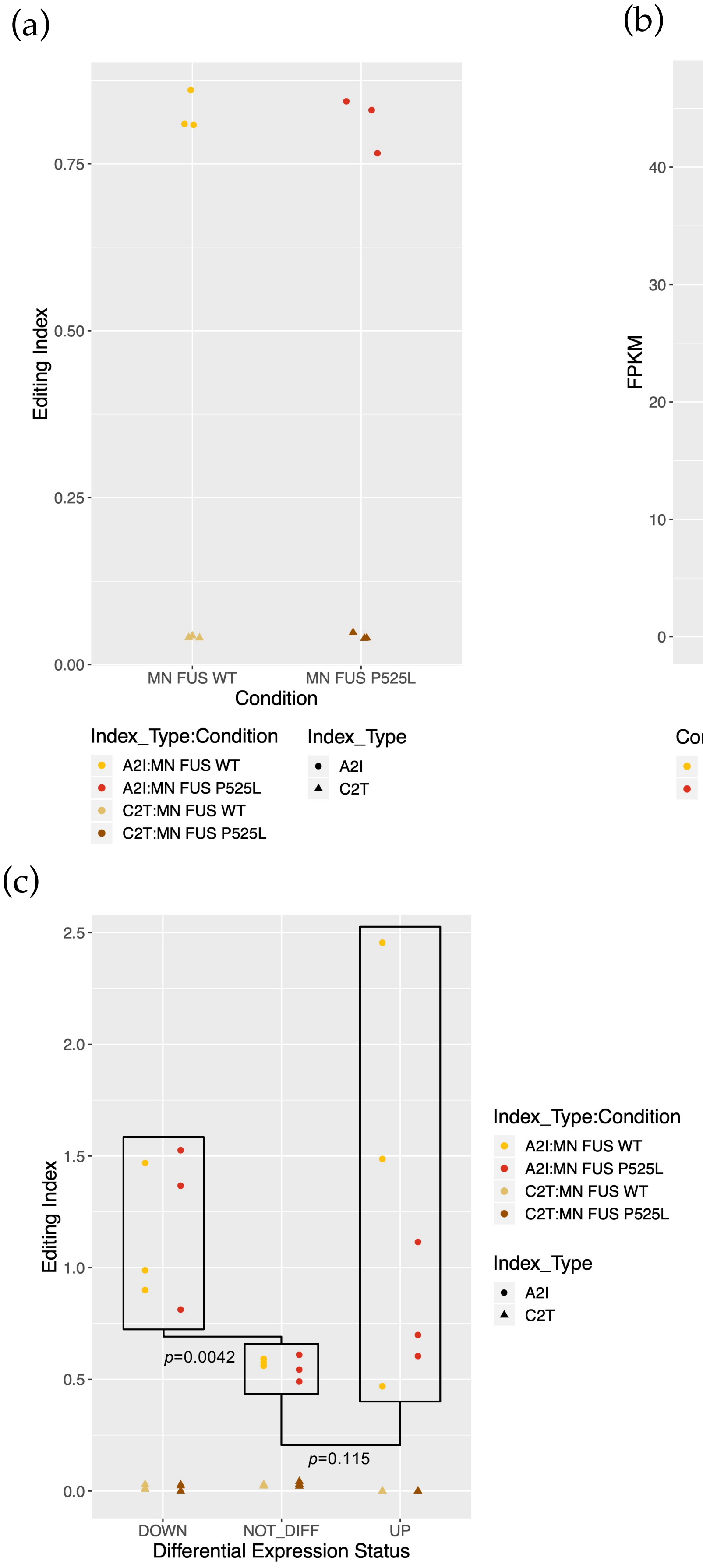

Figure S3 (related to Figure 3). Alu editing index and ADAR activity in human MNs (a) Strip chart reporting the Alu editing (A2I) indices calculated on all the Alu elements in MN FUS^{WT} and FUS^{P525L} samples. The triangles show the C-to-T index, which is a measure of background noise. (**b**) Expression values (FPKM) of the three ADAR enzymes expressed in MN FUS^{WT} and FUS^{P525L} samples. **(c)** Strip chart reporting the Alu editing (A2I) indices calculated in MN FUS^{WT} and FUS^{P525L} samples on the Alu elements found in the 1 kb-long intronic regions flanking circRNAs which are downregulated (DOWN), upregulated (UP) or not altered (NOT_DIFF) upon FUS mutation. The triangles show the C-to-T index, which is a measure of background noise. *p-values* were calculated by performing paired Student's t-tests.

Figure S4

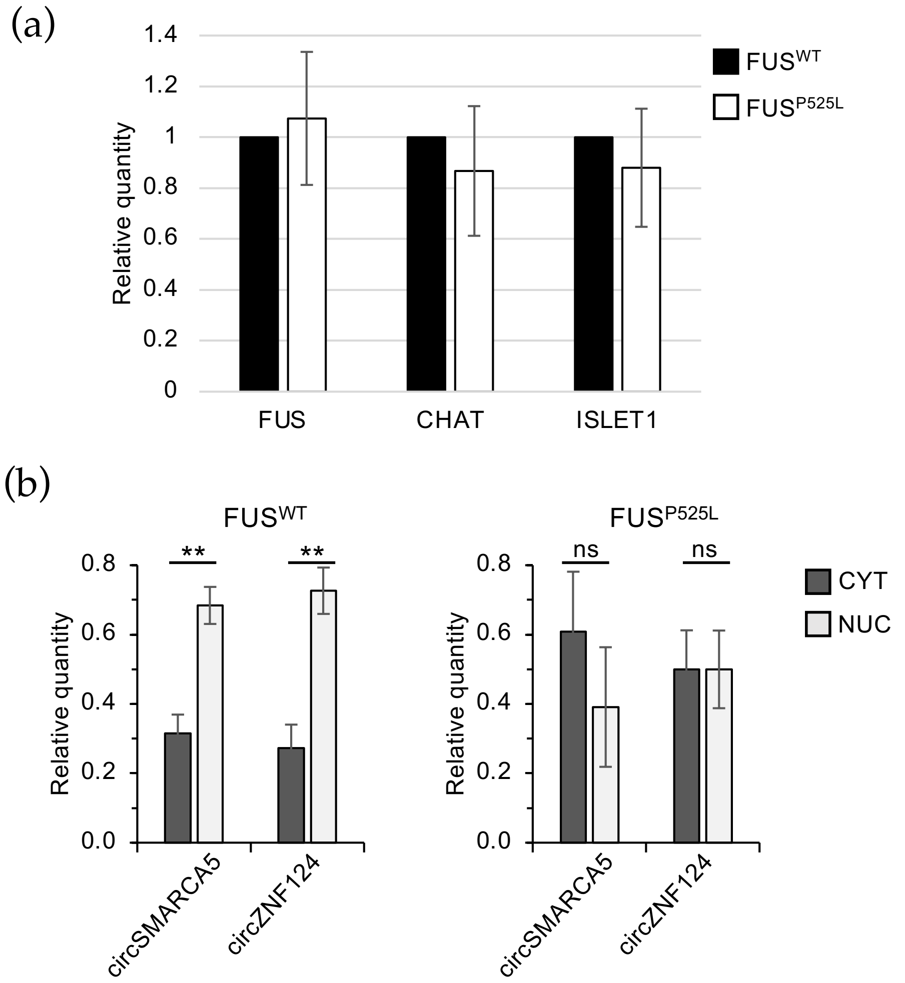

Figure S4. (Related to Figures 4 and 5) Expression of different genes in mature MNs. (**a**) Bar plot showing the levels of the indicated mRNAs measured by quantitative Real-Time PCR in FUS^{WT} and FUS^{P525L} mature MNs. Different mRNA levels were quantified using GAPDH mRNA as reference and expressed as relative quantity with respect to FUS^{WT} samples set to a value of 1. Error bars represent s.d. of three independent experiments. (**b**) Bar plot showing the levels of circSMARCA5 and circZNF124 measured by quantitative Real-Time PCR in nuclear (NUC) and cytoplasmic (CYT) compartments of FUS^{WT} and FUS^{P525L} mature MNs. Error bars represent s.e.m. of at least three independent experiments. p-values were calculated using two-tailed Student's *t-test* (**p<0.01).

Figure S5

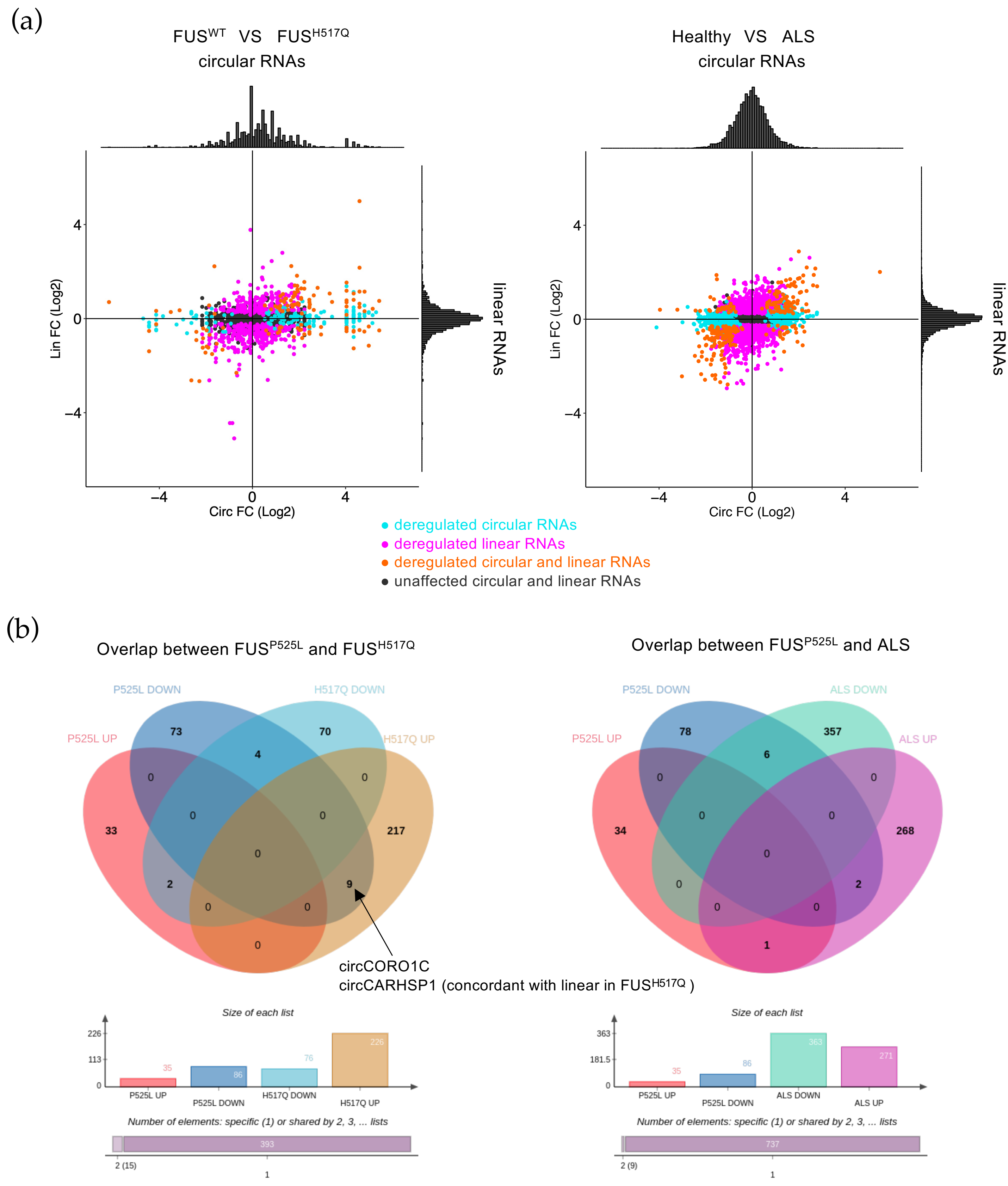

Figure S5. Differential expression of circular RNAs in other forms of ALS. (**a**) Scatter plots showing the change in the expression of circRNAs (x axis) and their cognate linear RNAs (y axis) in FUS^{H517Q} MNs with respect to FUS^{WT} MNs and in the spinal cord of ALS patients with respect to healthy controls [62,63]. The distributions of the log2(FC) values are shown above and aside the scatter plots. Turquoise dots are used to indicate when only the circRNA is deregulated (p-value < 0.05); magenta dots when only the linear RNA is deregulated (p-value < 0.1); orange or black dots when both the circRNA and the linear counterpart are deregulated or unaffected, respectively. (**b**) Venn diagrams showing the overlap between the upregulated (UP) and downregulated (DOWN) circRNAs identified in FUS^{P525L} MNs and those detected in FUS^{H517Q} MNs [62] and in the spinal coord of ALS patients [63]. The two circular RNAs deregulated both in FUS^{P525L} and FUS^{H517Q} MNs and validated in the present study are indicated.

Figure S6

GENE	OLIGO FORWARD	OLIGO REVERSE
**CARHSP1_circ**	AGGAGTCTGCAAATGCTTCT	AGCCCGACTGAAGCTTGATG
**CARHSP1_lin**	CARHSP1_circ Oligo Forward	ATTCTTGGGTGGGATGGAGC
**RHOBTB3_circ**	TCAGTGGGAAGAATTGGAAGA	ACACACTGGCAGCAGAACAG
**RHOBTB3_lin**	AATGAGCAACTCCTTTCATGG	RHOBTB3_circ Oligo Reverse
**CORO1C_circ**	CGGATGAATCCCCGTACGTC	AATGATGCTGGTGTCAGGGT
**CORO1C_lin**	GGAGAAAGCACATGAAGGAGC	CORO1C_circ Oligo Reverse
**ZNF124_circ**	ACTGTGAATGTAAGGTGTAGGAA	TCCAACAAAGCCCACTCCTC
**ZNF124_lin**	CTGTGA CCTGCATGTACTGG	Znf124_circ Oligo Reverse
**SMARCA5_circ**	CTCCAAGATGGGCGAAAG	TGTGTTGCTCCATGTCTAAT
**SMARCA5_lin_**	ACAGTCAGAGTGTTCCGCTT	TCTGATCCACAAGCCTCCCT
**NAA35_circ**	TTCCACCTACCTTCCCTCGA	TCAGTTCAGGCAAGGTGAGA
**NAA35_lin**	GCTGGCATGATTGGAAACCA	NAA35_circ Oligo Reverse
**PSME3_circ**	CGCACCGTGACAGAGATTGA	GACACCCCAAAGTTGTTTCCA
**PSME3_lin**	TGAAACCTGAGATCCGGCTG	PSME3_circ Oligo Reverse
**SLC8A1_circ**	CATCGAAGGGACTGCCAGAG	GGTGAAAGACTTAATCGCCGC
**SLC8A1_lin**	TGTGCTTCCCACAGAAGATG	SLC8A1_circ Oligo Reverse
**DLC1_circ**	CACCCAGGAACCCACAGATA	AGGGTAAAGGAGATGGAACTTGA
**DLC1_lin**	ACTAACGGGGACGGACTTCT	DLC1_circ Oligo Reverse
**ZNF609_circ**	CCCAGGAACCATTGGATAAG	CAGAGGTAAGATAAGGTCGG
**GAPDH**	GGAAGGTGAAGGTCGGAGTC	TTACCAGAGTTAAAAGCAGCCC
**pre-GAPDH**	CTGGGGGTAAGGAGATGCTG	TTACCAGAGTTAAAAGCAGCCC
**ATP5O**	ACTCGGGTTTGACCTACAGC	GGTACTGAAGCATCGCACCT
**ISLET1**	TACAAAGTTACCAGCCACC	GGAAGTTGAGAGGACATTGA
**CHAT**	TCATTAATTTCCGCCGTCTC	GAGTCCCGGTTGGTGGAGT
**FUS**	TCAGCTAAAGCAGCTATTGACTGG	GCCACCACCCCGATTAAAGTCTGC
**si-DLC1 #1**	GUAAGAGAAAGGUGGUGGUGAAUU, Dharmacon™	
**si-DLC1 #2**	AGAGAAAGGUGGUGAAAUGUUUU, Dharmacon™	
**scr-siRNA**	ON-TARGETplus Non-targeting Control siRNAs D-001810-01-20, Dharmacon™	
**ENAH**	CACCCTTATCACAGCCCAGT	TGCTCAGTTCCTGCCTGATT

Figure S6. List of Oligonucleotides used in this study.
